# Numerical investigation of the vortex-induced vibration of an elastically mounted circular cylinder at high Reynolds number (Re = 10^4^) and low mass ratio using the RANS code

**DOI:** 10.1371/journal.pone.0185832

**Published:** 2017-10-05

**Authors:** Niaz Bahadur Khan, Zainah Ibrahim, Linh Tuan The Nguyen, Muhammad Faisal Javed, Mohammed Jameel

**Affiliations:** 1 University of Malaya, Department of Civil, Faculty of Engineering, University of Malaya, Kuala Lumpur, Malaysia; 2 PetroVietnam, Exploration and Production Company, Hanoi, Vietnam; 3 Department of Civil Engineering, COMSATS Institute of Information Technology, Abbottabad, Pakistan; 4 Civil Engineering Department, Lords Institute of Engineering & Technology, Hyderabad, India; Abdul Wali Khan university Mardan Pakistan, PAKISTAN

## Abstract

This study numerically investigates the vortex-induced vibration (VIV) of an elastically mounted rigid cylinder by using Reynolds-averaged Navier–Stokes (RANS) equations with computational fluid dynamic (CFD) tools. CFD analysis is performed for a fixed-cylinder case with Reynolds number (Re) = 10^4^ and for a cylinder that is free to oscillate in the transverse direction and possesses a low mass-damping ratio and Re = 10^4^. Previously, similar studies have been performed with 3-dimensional and comparatively expensive turbulent models. In the current study, the capability and accuracy of the RANS model are validated, and the results of this model are compared with those of detached eddy simulation, direct numerical simulation, and large eddy simulation models. All three response branches and the maximum amplitude are well captured. The 2-dimensional case with the RANS shear–stress transport k-w model, which involves minimal computational cost, is reliable and appropriate for analyzing the characteristics of VIV.

## Introduction

Vortex-induced vibration (VIV) is a popular research topic in offshore engineering and fluid–structure interaction. This topic elicited the attention of researchers after the dramatic collapse of the Tacoma Narrows Bridge in 1940. The worldwide trend toward deep-sea oil production has also motivated researchers to analyze the VIV of cylindrical structures, such as risers, pipes, and marine cables. Chimney stacks, marine structures, and bridges may collapse or be damaged by the VIV phenomenon. The impact of different factors that affect VIV should be analyzed.

VIV analysis is performed with empirical tools, through numerical simulations, or via experiments. Empirical tools depend on the experimental data for the prediction and analysis of VIV or on numerical simulation method, which solves the Navier—Stokes equations to compute the forces on oscillating cylinder. With the advancement in computer technologies, the trend toward numerical simulation has increased exponentially in the last decade, and research on VIV has shifted to computational fluid dynamic (CFD) techniques. Although CFD analysis still presents several limitations, such as complex flow dynamics, high computational cost for 3-dimensional real problems, selection of a proper turbulent model. However, continuous advancement in computational capabilities, proper simplification techniques and the availability of comparatively inexpensive turbulent models increase the reliability and accuracy of numerical simulation tools. The reviews conducted by Breuer [1], Sarpkaya [2] and Bearman [3] elucidated the constraints and restrictions involved in numerical simulations. Turbulent properties of the flow are primarily solved using Large-eddy simulation, Reynolds-averaged NS and Direct-numerical simulations. DNS and LES are useful in assessing the wake–boundary layer interaction, but they are computationally expensive and require high computing power. Using the DNS technique at high Re is impractical due to the unrealistic computational power demand. Compared with LES and DNS, the RANS technique is more reliable and less time consuming when used to study VIV at high Re.

Numerous studies have investigated VIV in circular cylinders, but most of them involved low Re and low mass ratios. The 2-dimensional RANS code was utilized by Placzek et al. [4] to study VIV at a low mass ratio and low Re (Re = 100). The mode of vortex shedding was efficiently captured by the 2-dimensional RANS code at Re = 100. Zhao et al. [5] analyzed the VIV phenomenon with range of Reynolds number Re = 150 to 1000 using 3-dimensional Navier—Stokes equations. The authors concluded that 2-dimensional Navier—Stokes equations are inappropriate to analyze VIV behavior in a turbulent regime, whereas the 2-dimensional RANS equation performs well in this aspect. Niaz et.al [6] used LES code along the Smagorinksy—Lilly subgrid-scale model to analyze the wake characteristics and hydrodynamic coefficients of fixed structure at Re = 3900. One of the main targets of the simulations was to analyze the impact of the spanwise length and mesh resolution on calculating recirculation length and angle of separation. Islam, Manzoor, and Zhou [7] numerically studied the factors responsible for reduction in vortex shedding and corresponding drag forces at Reynolds number = 80 to 200 for flow around a square cylinder. Islam et.al [8] used the incompressible Boltzmann method to analyze the impact of aspect ratio of rectangular cylinder on vortex mode, fluid forces, and vortex-shedding frequency at Re = 100 to 250. Behara and Sotiropoulos [9] investigated the dynamic and wake modes of a sphere under VIV at Re = 300 to 100 at a low mass ratio. The study was conducted for reduced velocity (U_r_) ranging from 0 to 13. The authors concluded that the wake modes and trajectories of the sphere strongly depend on Re. Liangjie et al. [10] investigated VIV at different Re with the same shear flow parameter and discovered that the multimodal phenomenon is more significant at high Re. The dominant vibration mode was observed at the maximum order mode of natural frequency. The study of Tutar and Holdo [11] was based on LES with 2-dimensional and 3-dimensional models at Re = 24,000. In case of two dimensional simulations, authors observed deficiency in value of recirculation length, base pressure and drag coefficient. Dong and Karniadakis [12, 13] studied the flow for oscillating and fixed cylinders at Re = 24,000 by using the 3-dimensional DNS code. Detailed experimental study has been conducted by Khalak and Williamson [14] to investigate the VIV behavior for low mass ratio having range of Reynolds number Re = 1,700 to 12,000. The study was validated numerically by Pan and Cui [15], Wei Li et al. [16], and Guilmineau and Queutey [17]. Despite the agreement in predicting the vortex-shedding mode and the transition among different modes, these numerical studies are insufficient in terms of computing the maximum amplitude. These numerical simulations were performed on 2-dimensional models using different RANS codes. Nguyen and Nguyen [18] analyzed the VIV phenomenon at high Reynolds number and low mass ratio by using a hybrid of RANS and LES [also term as detached-eddy simulation (DES)]. The results obtained from DES study agreed well with the experimental results of Hover [19]. All numerical simulations performed in VIV literature at high Re were conducted using expensive 3-dimensional turbulent models, namely, DNS, LES, and DES.

The main objective of the current study is to check the accuracy and reliability of the RANS shear–stress-transport (SST) k-w turbulent model in predicting the VIV phenomenon of a circular cylinder at Re = 10^4^ and mass ratio (m* = 11). The results from this work are compared with the 3-dimensional DES study [18] and experimental results [19]. ANSYS FLUENT 16 is used for all numerical simulations. This work is expected to provide a path for analyzing and predicting the VIV phenomenon in a supercritical region (3.5 × 10^5^ < Re < 1.5 × 10^6^).

## Numerical approach

In current study, the flow is assumed to be incompressible in nature. The unsteady RANS equation can be written as:

$$\frac{\partial {\text{u}}_{\text{i}}}{\partial {\text{x}}_{\text{i}}}=0$$ (1)

$$\frac{\partial }{\partial \text{t}}({\rho \text{u}}_{\text{i}})+\frac{\partial }{\partial {\text{x}}_{\text{j}}}({\rho \text{u}}_{\text{i}}{\text{u}}_{\text{j}})=-\frac{\partial \rho }{\partial {\text{x}}_{\text{i}}}+\frac{\partial }{\partial {\text{x}}_{\text{j}}}\left(2\mu {\text{S}}_{\text{ij}}-\rho \bar{{\text{u}}_{\text{i}}^{\prime }{\text{u}}_{\text{j}}^{\prime }}\right)$$ (2)

where ρ and u_i_ are time-average values of pressure and velocity, respectively; μ represents molecular viscosity; and S_ij_ and $\;\bar{{\text{u}}_{\text{ij}}}$ are the mean stress tensor and Reynolds stress tensor, respectively. All of these can be solved by a Newtonian model as follows:

$$-\rho \bar{{\text{u}}_{\text{i}}^{\prime }{\text{u}}_{\text{j}}^{\prime }}={\text{u}}_{\text{i}}\left(\frac{\partial {\text{u}}_{\text{i}}}{\partial {\text{x}}_{\text{j}}}+\frac{\partial {\text{u}}_{\text{j}}}{\partial {\text{x}}_{\text{i}}}\right)-\frac{2}{3}\left(\rho \text{k}+{\text{u}}_{\text{i}}\frac{\partial {\text{u}}_{\text{i}}}{\partial {\text{x}}_{\text{j}}}\right)\delta_{\text{ij}}$$ (3)

where eddy viscosity μ_i_, which is a scalar property, is usually computed from a transport variable; δ_ij_ is the kronecker delta; and turbulent kinetic energy k can be presented as:

$$\text{k}=\frac{\bar{{\text{u}}_{\text{i}}^{\prime }{\text{u}}_{\text{j}}^{\prime }}}{2}=\frac{1}{2}\left(\bar{{\text{u'}}^{2}}+\bar{{\text{v'}}^{2}}\right)$$ (4)

This model was further explained in detail by Menter [20].

The unsteady segregated algorithm is adopted in the calculation. Pressure–velocity coupled equations are solved with the SIMPLE algorithm [explained in the ANSYS manual [21]], and the implicit 1st-order scheme is utilized for unsteady terms. The 2nd-order scheme is used for k-ω transport equations and for convection terms in the momentum equations. The upwind scheme, which is of the first order, is applied to the diffusion terms.

Guilmineau and Queutey [17] indicated that the dimensionless transverse displacement of a circular cylinder under VIV can be presented as:

$$\frac{{\text{d}}^{2}\text{Y}}{\text{d}\tau^{2}}+\frac{4\pi \zeta }{{\text{U}}_{\text{r}}}\frac{\text{dY}}{\text{d}\tau }+\frac{4\pi^{2}}{{\text{U}}_{\text{r}}{}^{2}}\text{Y=}\frac{2{\text{C}}_{\text{y}}}{\pi \text{m}*}$$ (5)

where Y = y/D represents the displacement in the transverse direction normalized by the cylinder diameter; U_r_, ζ, m*, and C_y_ are the reduced velocity, structural damping ratio, mass ratio, and life coefficient, respectively.

At the lock-in region, the vortex-shedding frequency approaches the cylinder oscillating frequency and the following equations prevail.

$$\text{y}=\text{Asin}({\text{w}}_{\text{ex}}\text{t})$$ (6)

$${\text{C}}_{\text{y}}={\text{C}}_{\text{L}}\text{sin}({\text{w}}_{\text{ex}}\text{t}+\emptyset )$$ (7)

$${\text{C}}_{\text{Lv}}={\text{C}}_{\text{L}}\text{sin}\emptyset$$ (8)

$${\text{C}}_{\text{La}}=-{\text{C}}_{\text{L}}\text{cos}\emptyset$$ (9)

where w_ex_ is the oscillating cylinder frequency and C_L_, C_La_, and C_Lv_ are the amplitude of the lift coefficient and its corresponding components of acceleration and velocity, respectively. Parkinson [22] indicated that the amplitude ratio (A* = A_y_/D) and frequency ratio (f* = f_ex_/f_n_) can be defined as:

$$\text{A}*=\frac{1}{4}\frac{{\text{C}}_{\text{Lv}}}{\pi^{3}\text{m}*m\zeta }\frac{{\text{f}}_{\text{n}}}{{\text{f}}_{\text{ex}}}{\left(\frac{\text{U}}{{\text{f}}_{\text{n}}\text{D}}\right)}^{2}$$ (10)

$$\text{f}*={\left[1+\frac{1}{2}\frac{{\text{C}}_{\text{La}}}{\pi^{3}\text{m}*\text{A}*}{\left(\frac{\text{U}}{{\text{f}}_{\text{n}}\text{D}}\right)}^{2}\right]}^{\frac{1}{2}}$$ (11)

Eqs (10) and (11) are derived from the linearization with energy balance between the cylinder and the fluids.

The other dimensionless terms used in this study are defined below:

1. Mass ratio
  The ratio between oscillating cylinder mass (m_osc_) and the displaced fluid mass (m_f_) is defined as mass ratio and is given by.
  $$\text{m}*=\frac{{\text{m}}_{\text{osc}}}{{\text{m}}_{\text{f}}}$$ (12)
  and ${\text{m}}_{\text{f}}=\frac{1}{4}\rho \pi {\text{D}}^{\text{2}}\text{L}$
  where ρ, D and L represent the density of fluid, diameter of cylinder and submerged length of cylinder, respectively.
2. Reynolds number (Re)
  The ratio of inertial force to viscous force within the fluid is known as Reynolds number and is given by;
  $$\text{Re}=\frac{\text{UD}}{\upsilon }$$ (13)
  Where U, D and υ represent the inlet velocity, characteristics length and kinematic viscosity, respectively
3. Strouhal number (St)
  The vortex-shedding frequency of a fixed cylinder is defined by a non-dimensional quantity known as the Strouhal number (St).
  $$\text{St}=\frac{{\text{f}}_{\text{v}}\text{D}}{\text{U}}$$ (14)
  where f_v_, D and U represent vortex-shedding frequency, diameter of a cylinder and current velocity of the fluid. In the current study, Fast Fourier Transform (FFT) algorithm has been utilized to determine the Strouhal number.
4. Reduced velocity
  Reduced velocity (U_r_), which is mean velocity normalized by diameter of cylinder and natural frequency of structure, is an important parameter when the structure begins oscillation due to the VIV phenomenon. This parameter is defined by
  $${\text{U}}_{\text{r}}=\frac{\text{U}}{{\text{Df}}_{\text{n}}}$$ (15)
  Where U, D and f_n_ are velocity of fluid, diameter of cylinder and natural frequency of structure, respectively

## Computational domain and mesh

**Fig 1:**
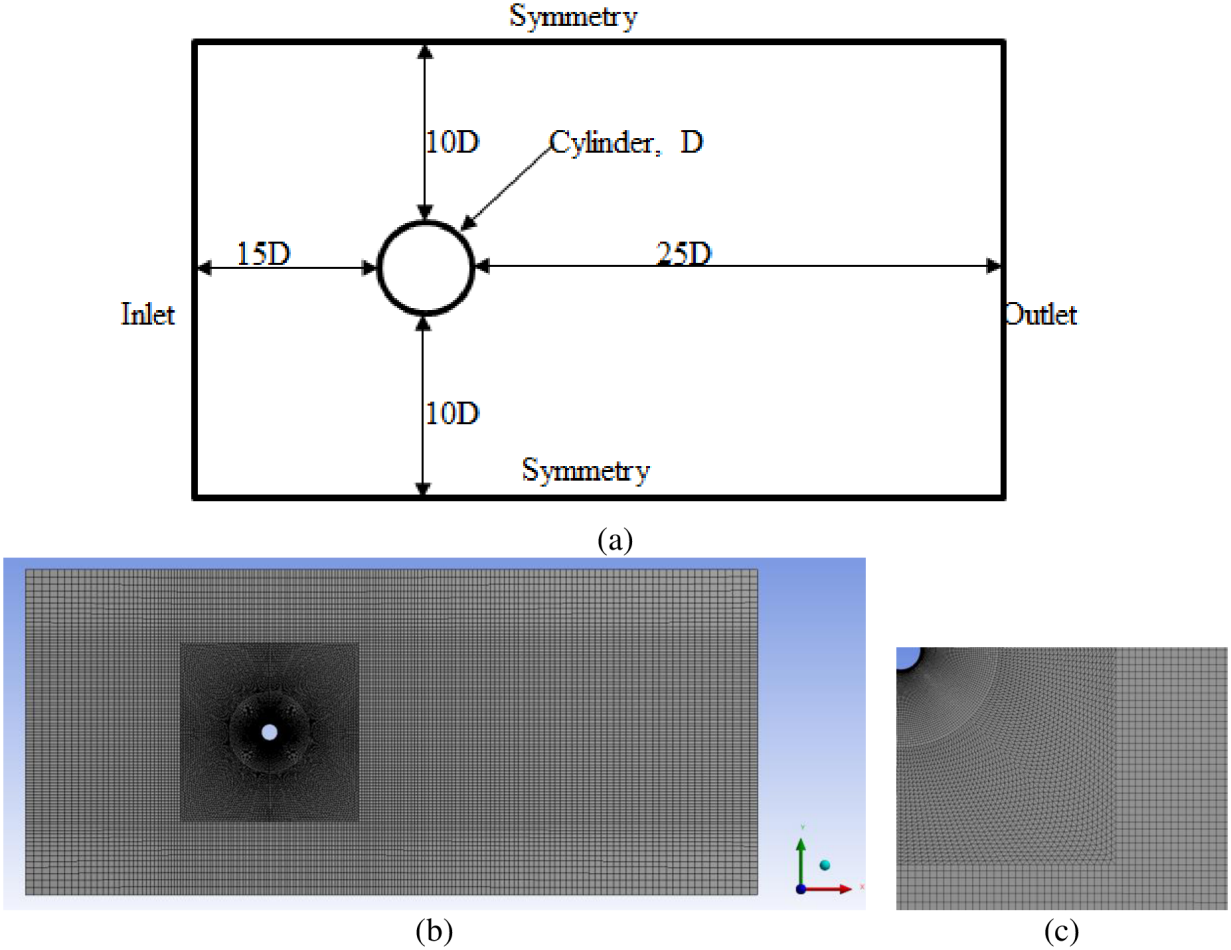
Computational domain and mesh (a) Geometry with boundary conditions (b) Mesh (c) Mesh detail section view near cylinder.

The size of domain has significant impact on the behavior of the flow, both in the steady and unsteady state. Various domain sizes have been used in literature. Shao [23] used the domain size of 30D × 16D for flow around cylinder study using RANS code. In the numerical investigation of the hydrodynamic performance, a domain size of 8D was adopted in the transverse direction by Fang and Han [24]. Domain sizes of 25D × 20D and 27D × 9D were used by Franke and Frank [25] and Li [24], respectively. In the current work, a computational domain size of 45D × 20D is used (Fig 1). Based on previous studies, in which a smaller domain was selected, the current domain size is sufficiently large to avoid the disturbance caused by boundary conditions. Furthermore, 5% of the blockage ratio is assumed to be adequate to diminish the impact of boundary conditions on the flow field, as suggested by Zdravkovich [26].

In all case studies, a hybrid unstructured mesh is created in such a manner that the meshes are very fine around the wall of the cylinder and coarse at the far region. The distance between the first node and cylinder is important in extracting accurate results. According to ANSYS [21], the y+ value, which depends on the distance between the first node and cylinder wall, should be less than or equal to unity to ensure the adequate resolution of the grid near the cylinder in simulation. In the current study, y+ = 1 is maintained in all cases with U_r_ ranging from 3 to 14. The grid is divided in several zones with the condition that the grid is symmetric and smooth throughout the domain to ensure stability and convergence (Fig 1B and 1C). Grid independence tests are conducted at Ur = 5.84, m* = 11, ζ = 0.001, Re = 10000 for the results of the maximum cylinder response amplitude (A_y_/D) [Table 1]. Mesh sensitivity tests are conducted by varying the grid resolution at the cylinder wall and near-field grid. Results of maximum cylinder response amplitude is validated against the experimental [19] and numerical study [18]. Table 1 presents the results for six different meshes. It is observed that T3 is able to achieve acceptable value of maximum response amplitude and further refinement in the mesh have negligible impact on the results. Therefore, T3 is finalized for all the simulations in current study.

**Table 1:** Grid independence study with RANS SST k-w model (Ur = 5.84, m* = 11, ζ = 0.001, Re = 10000).

	Cylinder wall	Near-field grid	Total elements	Max A_y_/D	Experimental A_y_/D [19]	Numerical A_y_/D [18]
T1	120	7200	24220	0.8628	1.003	0.9230
T2	120	9600	28310	0.8453		
T3	240	7200	45720	0.9210		
T4	240	9600	52650	0.9216		
T5	360	7200	72640	0.9242		
T6	360	9600	77240	0.9242		

The inlet boundary, which is on the left side of the domain, is 15 D from origin of the cylinder; the outlet boundary, which is at the right side of the cylinder, is 30 D from the origin of cylinder. A uniform velocity of 0.3149 m/s is applied at the domain inlet, which corresponds to Re = 10^4^ (where D = 1 m, density = 1000 kg/m^3^, and viscosity = 0.03149 kg/m-s). In current study, all the important physical parameters like Re, m*, ζ and frequency ratio, are dimensionless in nature. Value of D, U, ρ, ʋ, k, m* and ζ are taken in such a way that the important non-dimensional parameters are are similar to those used in the experiments of Hover [19] and numerical simulations of Nguyen et al. [18]. An average static reference pressure of 0 Pa is applied at the outlet boundary. A symmetric condition is assigned to the lower and upper sides and maintained at a distance of 10 D from the cylinder center. No-slip condition is assigned to cylinder wall where velocity increases from zero at cylinder wall to the free stream velocity in the far region.

## Results and discussions

Numerical simulation is conducted initially for a fixed-cylinder case to test the capability of the mesh and RANS SST k-w with 2-dimensional and 3-dimensional models. The simulation is performed at Re = 10,000. After the fixed-cylinder case, VIV is analyzed with a single degree of freedom (DOF) at Reynolds number = 10^4^ and a mass ratio of 11. All physical parameters are the same as those used by Hover [19] and Nguyen [18] in their experimental and numerical studies, respectively. In all case studies, y+ value equal to unity is maintained. A non-dimensional time step of 0.005 is used in all case studies.

### Fixed cylinder case

**Fig 2:**
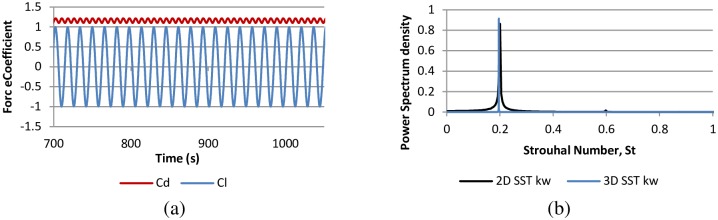
(a) Force coefficients and corresponding (b) Strouhal number for flow past fixed cylinder at Re = 10000.

Numerical simulations for flow around fixed cylinder is performed with two-dimensional and three-dimensional model at Reynold number Re = 10^4^ by using RANS SST k-w. The main purpose of these case studies is to test the RANS SST k-w model and the capability of the selected mesh to extract the results. The impact of spanwise length is reduced by assigning the periodic boundary conditions in the spanwise direction. Due to complex shape of cylinder, a variation in pressure distribution occurs on the cylinder surface due to the vortex-shedding phenomenon. The non-uniform pressure distribution along the cylinder wall results in a fluctuation in the lift forces acting on the cylinder, which leads to cross-flow oscillation and VIV. Table 2 presents a comparison between current results, experimental [27, 28] and numerical results [[13] [18] [29]] for the fixed-cylinder case. Drag coefficient is defined as C_d_ = 2F_d_ / (ρU^2^A), where F_d_ is the drag force in stream-wise direction integrated over cylinder surface and ρ, U and A are density of fluid, Inlet velocity and projected frontal area of the cylinder, respectively. Lift coefficient is defined as C_l_ = 2F_l_ / (ρU^2^A), where F_l_ is the lift force on the cylinder surface in the cross-flow direction. Calculations are based on Finite volume method (FVM). The drag and lift is calculated by integrating components (cells) values around the cylinder. The root-mean-square value of C_l_ is calculated from the time history data of C_l_ (Fig 2a). The time histories of C_d_, C_l_, and corresponding St obtained from the current simulations are shown in Fig 2. The C_d_ and St values are well computed by the RANS SST k-w model. However, a deficiency exists in computing the root-mean-square C_l_. The difference in the results is due to the impact of the aspect ratio, as investigated by Norberg [27] and Nguyen [18] in their experimental and numerical studies, respectively. The study suggested a high aspect ratio to obtain a low value of root-mean-square C_l_. Fig 2b shows the plot of power spectrum density of lift force coefficient history against St. Strouhal frequency computed using the SST k-w model agrees well with the experimental results.

**Table 2:** Comparison of C_d_, C_l_, and St at Re = 10,000 with numerical and experimental results available in literature.

	C_d, mean_	C_l.rms_	St
Norberg [27], Exp	-	0.25–0.46	0.202
Gopalkrishnan[28] Exp	1.19	-	0.193
Stephen et. al [29], LES	1.22	0.476	0.20
Nguyen [18], DES	1.133	0.262	0.2005
Dong et.al [13], DNS	1.143	0.448	0.203
2-dimensional- SST k-w	1.150	0.701	0.201
3-dimensional—SST k-w	1.210	0.646	0.203

**Fig 3:**
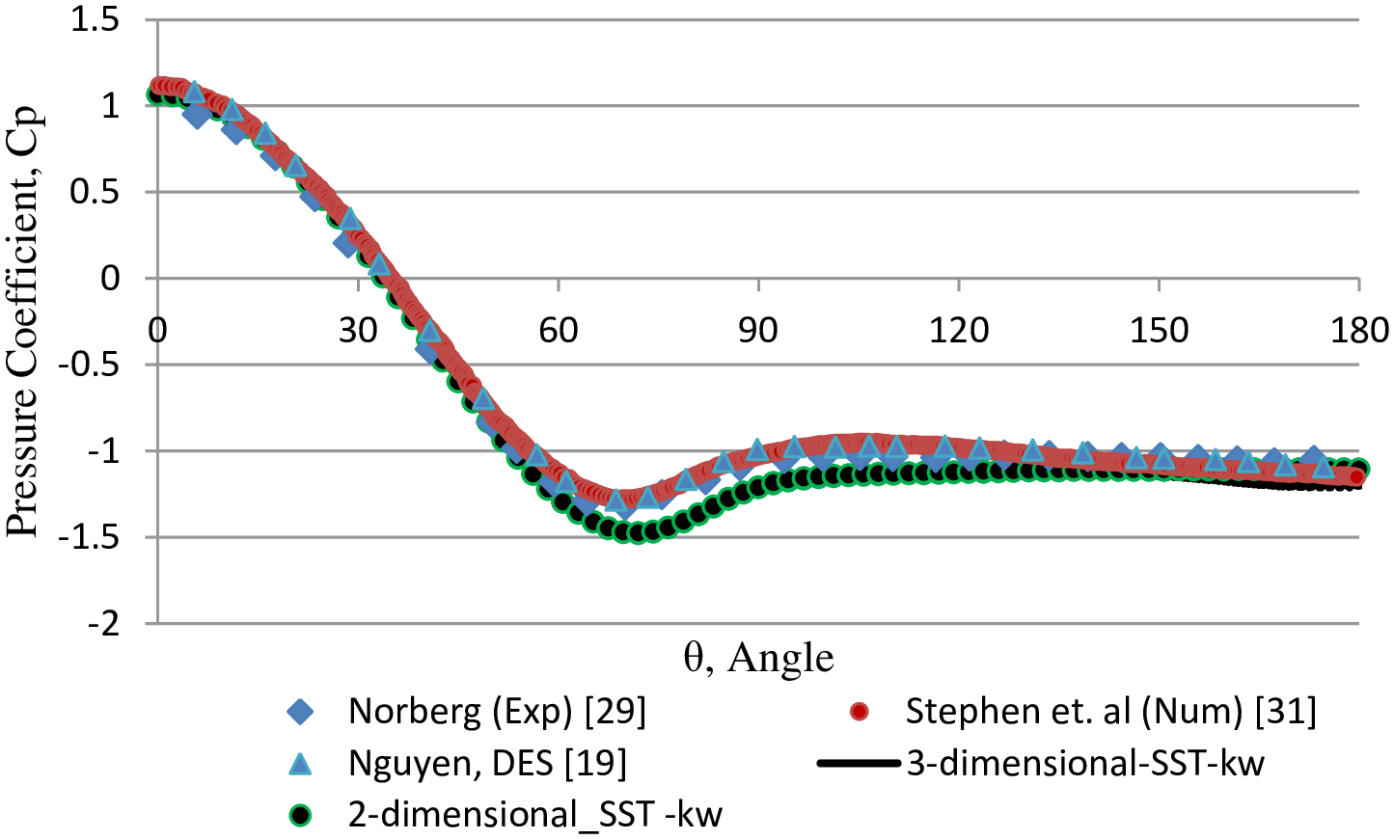
Comparison of mean pressure coefficient distribution at cylinder surface for fixed cylinder at Re = 10000.

**Fig 4:**
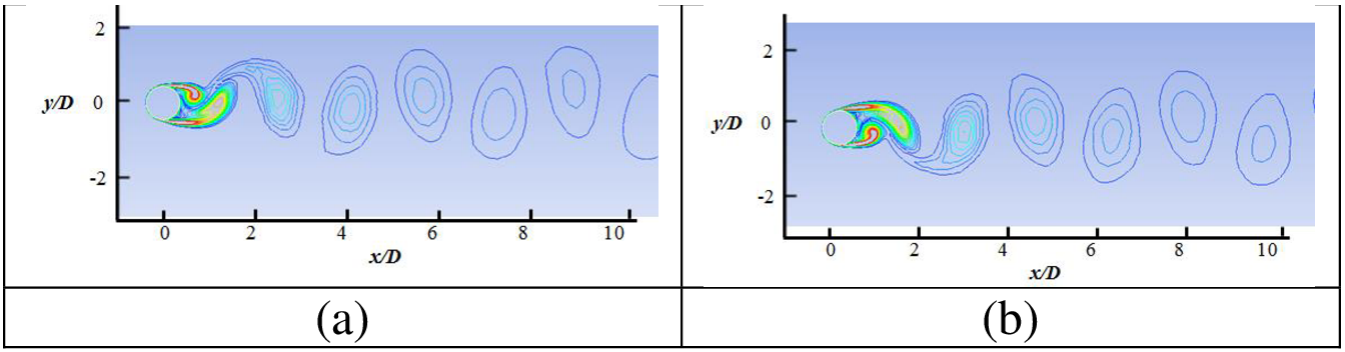
Instantaneous wake strutures at (a) maximum lift coefficeint and (b) minimum lift coefficient at Reynolds number Re = 10,000.

Fig 3 presents the plot of the mean pressure coefficient along the cylinder wall. In the numerical simulation, the pressure coefficient is calculated as C_p_ = 2P/(ρU^2^), where P, ρ, and U are the pressure, density, and uniform inlet velocity of fluid, respectively. Comparison of results depict a slight variation at the flow separation region (i.e., 70° to 80°), which shows the deficiency of the SST k-w model in extracting the results in this specific region. Fig 4 shows the detailed instantaneous wake structure behind the cylinder at maximum and minimum C_l_. Alternative vortex shedding from the upper and lower regions of the cylinder is observed at maximum and minimum C_l_, respectively, which results in periodic forces on the cylinder. These periodic forces are assumed to be responsible for the VIV phenomenon.

This comparison of results indicates that 2-dimensional SST k-w, which is less expensive than DNS [13], LES [29], and DES [18], can extract acceptable results at Re = 10,000. With this conclusion, numerical study is extended to analyze the VIV phenomenon in which cylinder is allow to oscillate in cross flow direction at Re = 10^4^ and mass ratio m* = 11.

### VIV analysis for smooth circular cylinder free to oscillate in the cross-flow direction (1 DOF)

**Fig 5:**
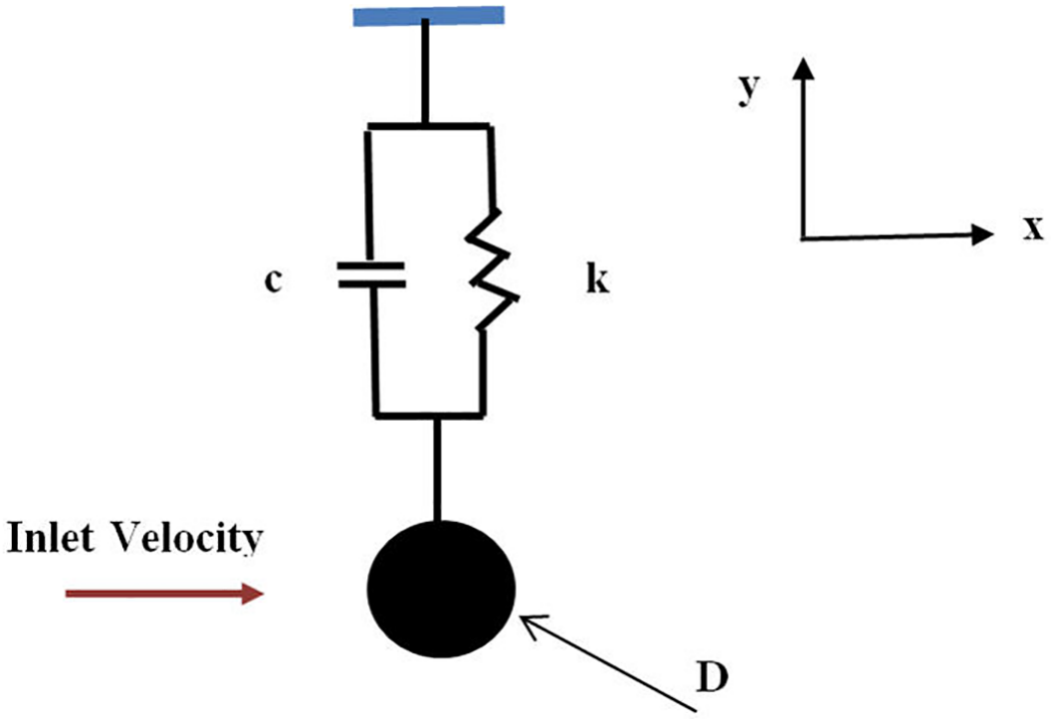
Flow around a cylinder with 1DOF.

After the numerical investigation of flow around a fixed cylinder, the study is extended to the case study in which cylinder is allow to oscillate in the y- direction, as shown in Fig 5. Cylinder oscillation is constrained in the y-direction by a spring-damper system with spring constant K and damping coefficient c. Analysis is performed at Re = 10^4^, damping ζ = 0.001, and mass ratio m* = 11. These parameters were also used in the experimental studies of Hover [19] and numerical simulation of Nguyen [18]. Numerical simulations are performed with U_r_ ranging from 3 to 14. The change in U_r_ is obtained by changing the frequency ratio w_t_ while maintaining the velocity. Hover [19] indicated that cylinder vibration is characterized by frequency ratio w_t_ (ratio of damped natural frequency to fixed-cylinder vortex-shedding frequency).

**Fig 6:**
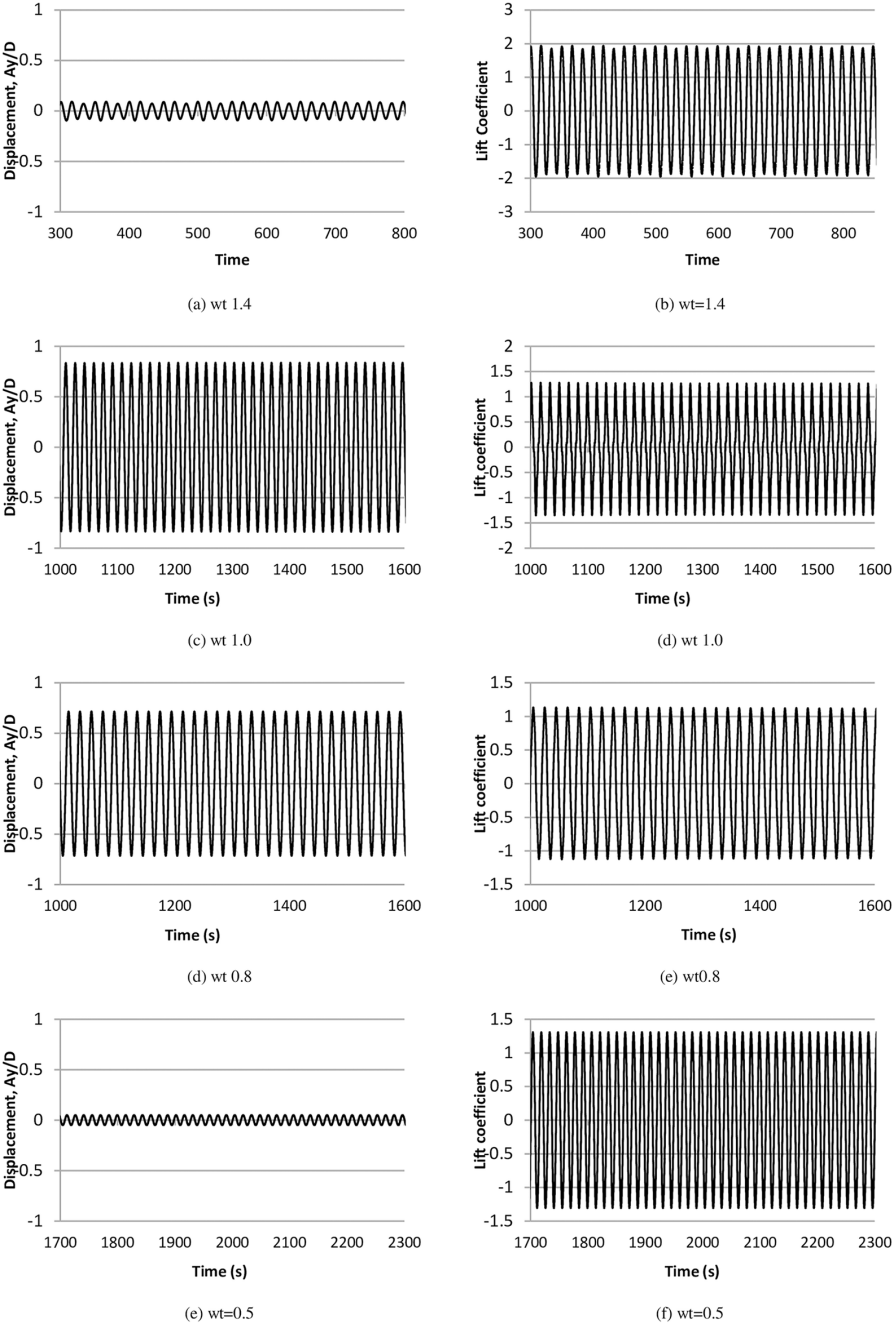
Time history of non-dimensional amplitude and lift coefficient at various ratio, (m* = 11, ζ = 0.001, Re = 10000).

**Fig 7:**
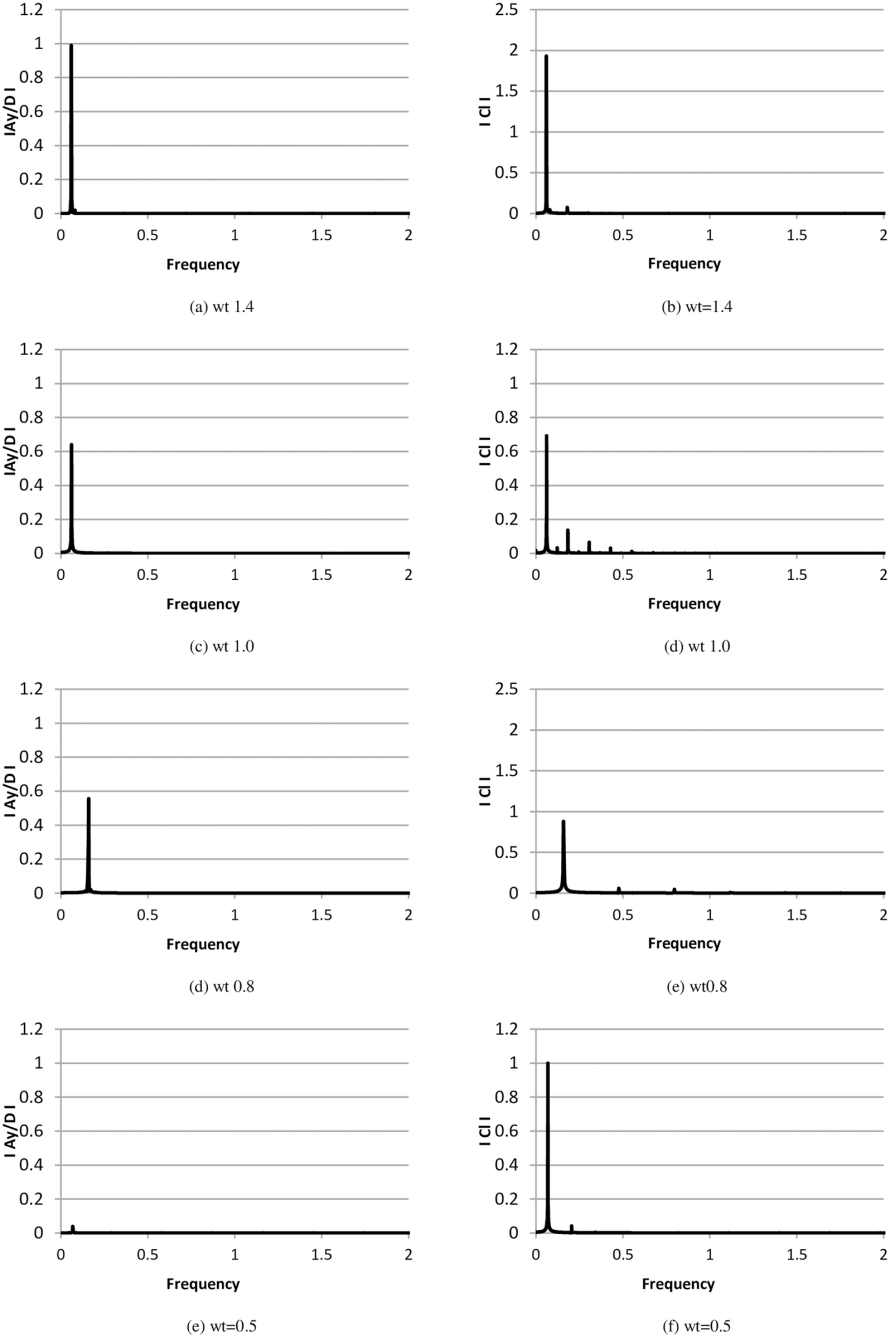
Power spectra non-dimensional amplitude and lift coefficient at Re = 10000 (m* = 11, ζ = 0.001).

**Fig 8:**
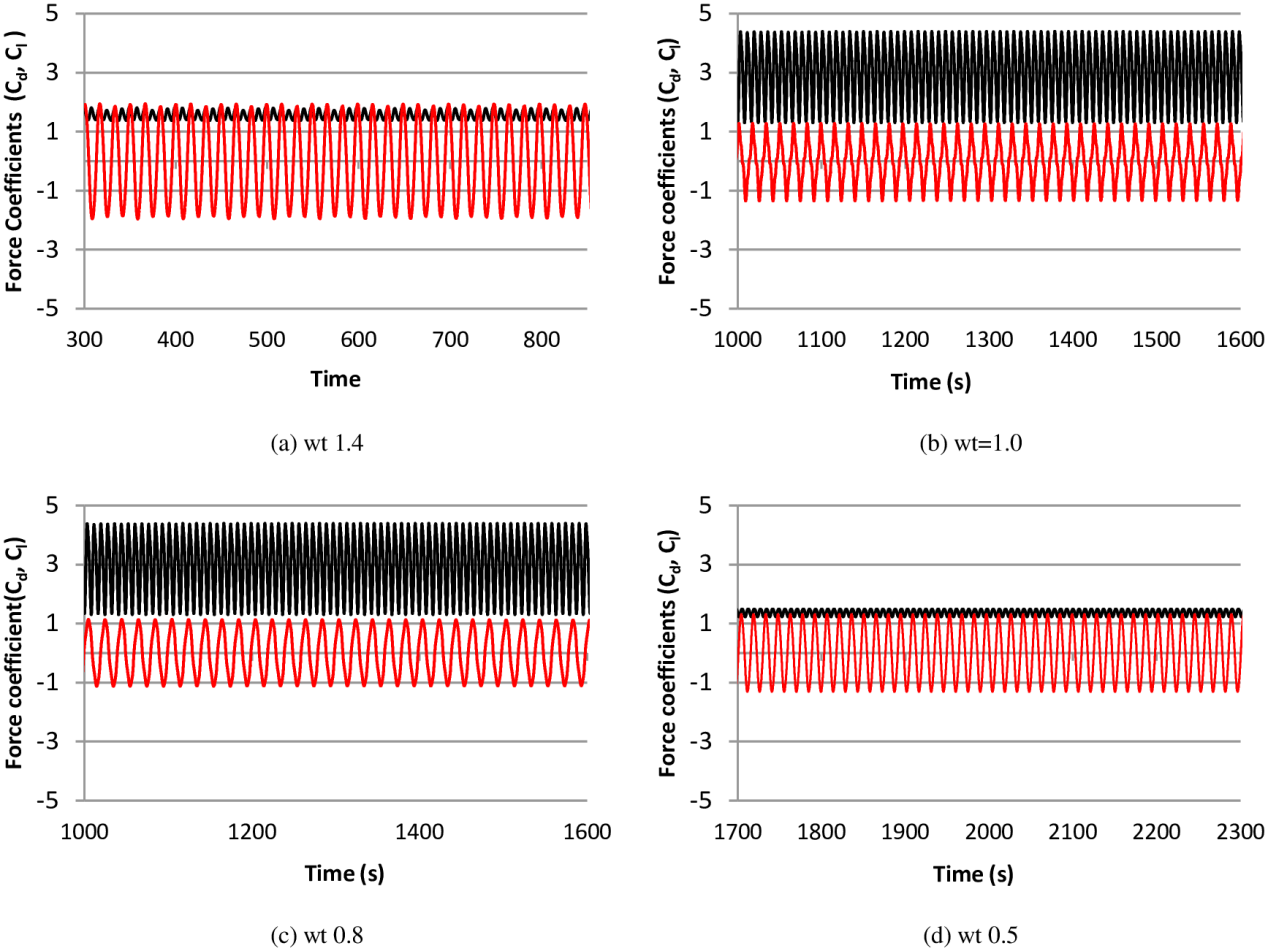
Time history of Cd (black-line) and Cl (red-line) at various frequency ratio, (m* = 11, ζ = 0.001, Re = 10000).

The time histories of non-dimensional cylinder amplitude and C_l_ at various frequency ratios are shown in Fig 6. The corresponding power spectra for cylinder amplitude and C_l_ are shown in Fig 7. When the natural frequency of the structure approaches the vortex-shedding frequency, the lock-in phenomenon or synchronization occurs, which in turn causes the cylinder oscillation to reach the maximum amplitude. This behavior is observed in Fig 6, in which the highest amplitude is found at frequency ratios wt = 1.0 and 0.8. A low value of cylinder response is also observed at wt = 1.4 and 0.5. Furthermore, single frequency in the cylinder displacement amplitude is observed, and the lift force coefficients have multiple peaks (Fig 7). Fig 8 compares the time history of the instantaneous C_d_ and C_l_ at various frequency ratios. It is observed that there is weak correlation between instantaneous C_d_ and C_l_, since the simulations is carried out using 2-dimensional model. In addition to the highest amplitude and multiple peaks in the power spectra of C_l_, high values of drag forces are also found in the upper branch, as shown in Fig 8b and 8c. Out of the lock-in region, comparatively small values of drag forces are observed. This behavior of drag forces has also been reported by Bishop and Hassan [30].

**Fig 9:**
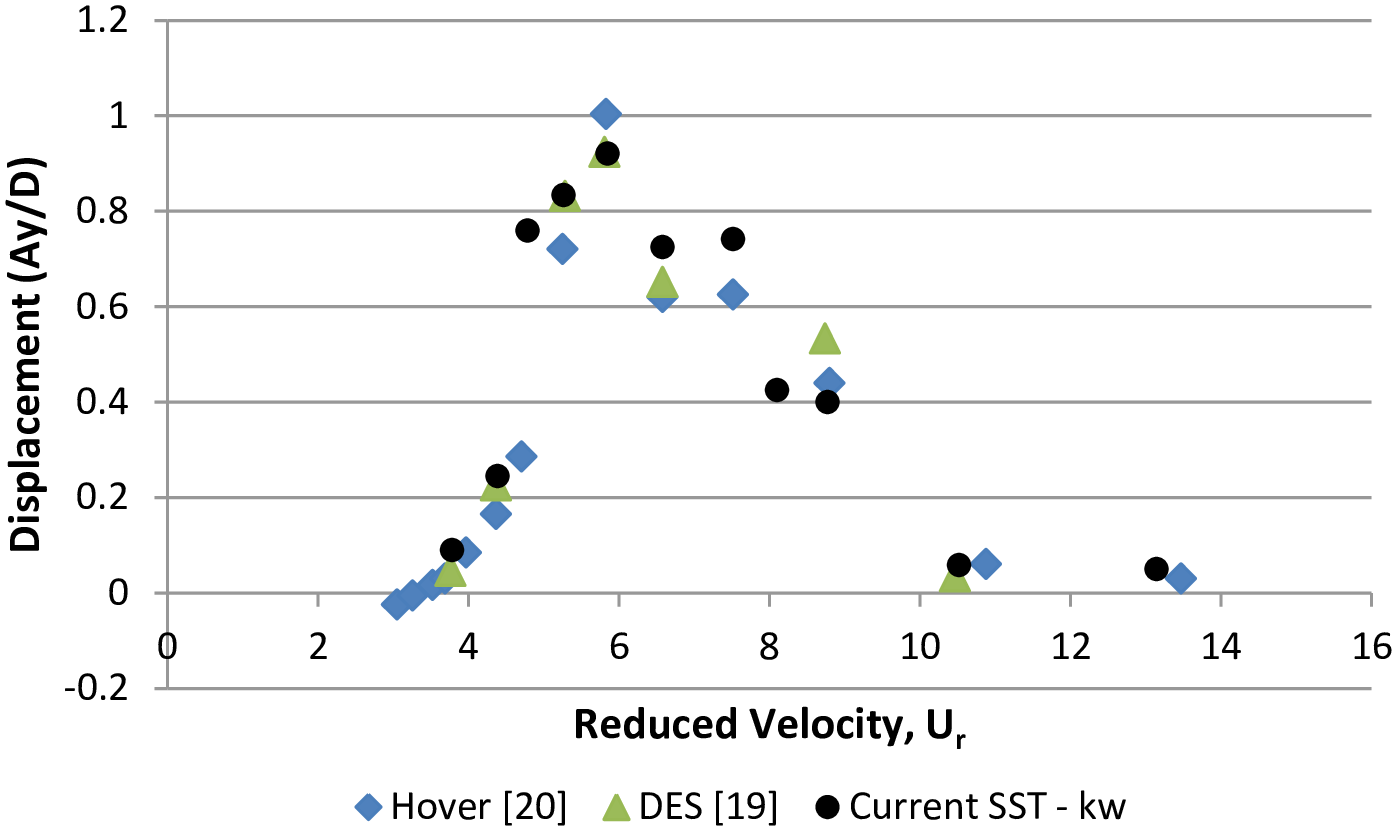
Comparison of cylinder response amplitude using the SST k-w model with experimental data and numerical DES data (m* = 11, ζ = 0.001, Re = 10000).

The non-dimensional amplitude of the cylinder computed in the current study is compared with that of a 3-dimensional DES study [18] and experimental results [19], as shown in Fig 9. Cylinder response is extracted from Fig 6, which shows the time history of cylinder transverse displacement. In all these cases, Re = 10^4^ is kept constant, and the change in Re is obtained by changing the frequency ratio. The cylinder response is categorized in three distinct branches according to classification of Khalak & Williamson [31] i.e. “initial branch”, “upper branch” and “lower branch. All the three types of response are well captured by SST k-w model as shown in Fig 9. Similar to the 3-dimensional DES approach, the SST k-w model also reveals the transition between the initial and upper branches at U_r_ = 4.7. The maximum amplitude response of Ay/D = 0.92 is observed in the upper branch at U_r_ = 5.84 (wt = 0.9), which agrees well with the 3-dimensional DES approach. However, comparatively early transition at U_r_ = 8.1 (w_t_ = 0.65) is observed between the upper and lower branches. Comparison of the results shows good agreement among the results of the current SST k-w model, DES [18], and Hover [19].

**Fig 10:**
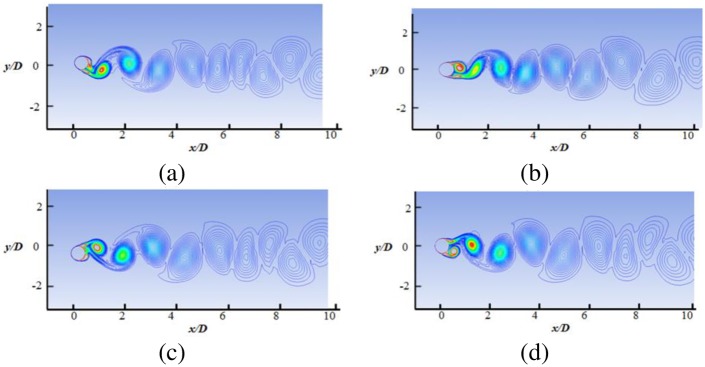
Vorticity contour at Ur = 3.78 (m* = 11, ζ = 0.001, Re = 10000).

**Fig 11:**
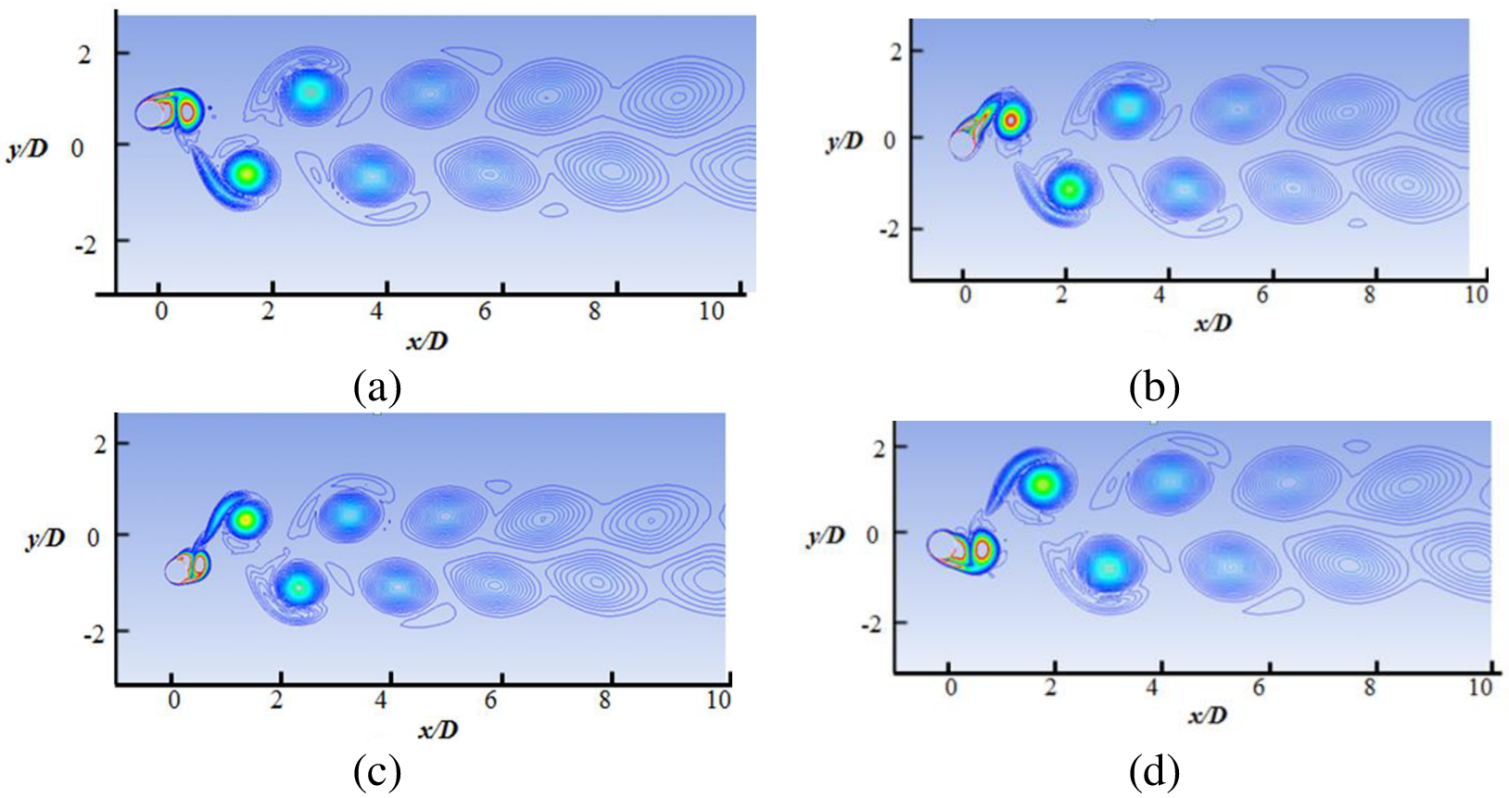
Vorticity contour at Ur = 5.84 (m* = 11, ζ = 0.001, Re = 10000).

**Fig 12:**
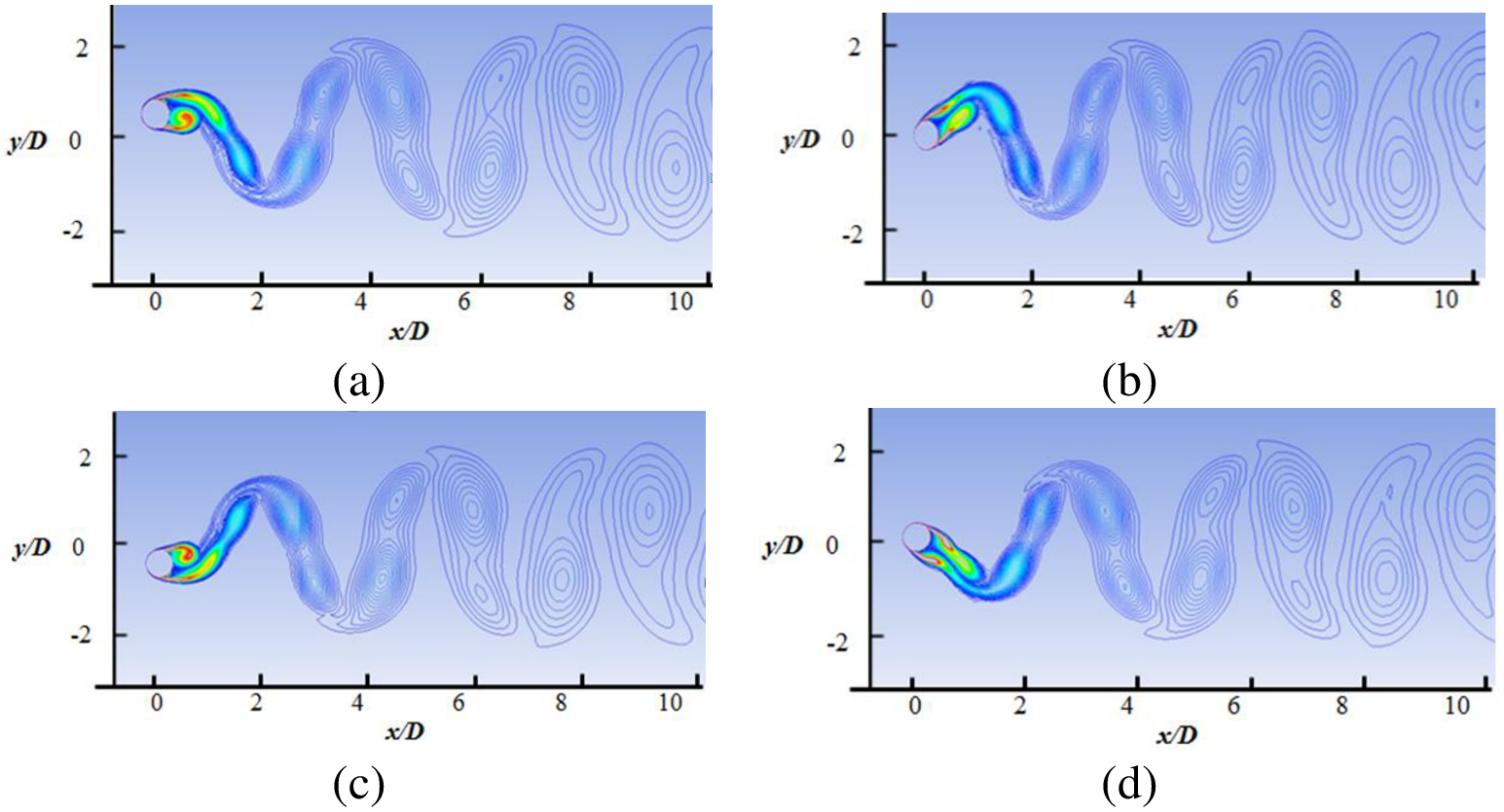
Vorticity contour at Ur = 7.52 (m* = 11, ζ = 0.001, Re = 10000).

**Fig 13:**
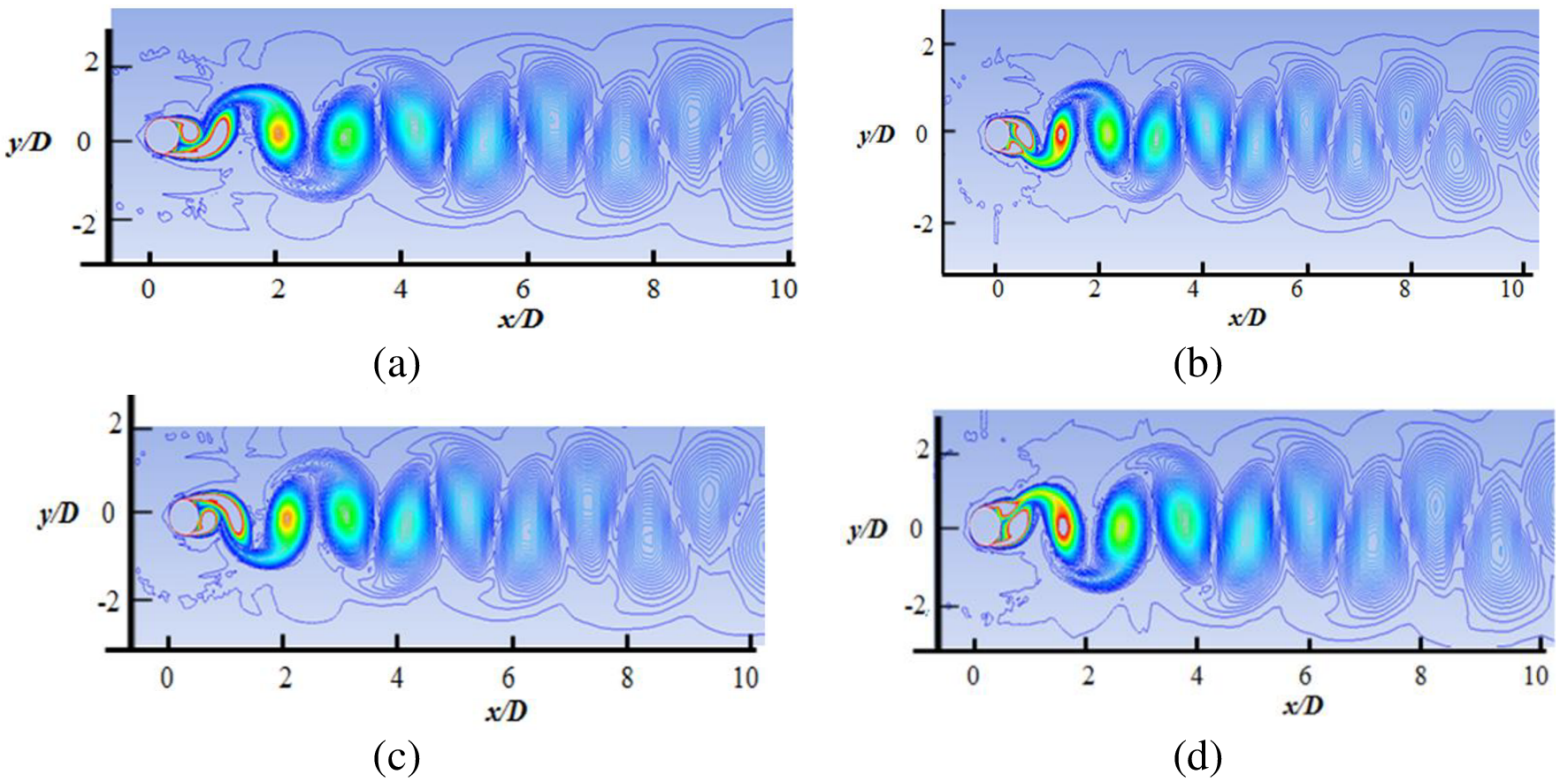
Vorticity contour at Ur = 8.77 (m* = 11, ζ = 0.001, Re = 10000).

The details of the wake structures behind the cylinder are shown in Figs 10 to 13, for reduced velocity Ur = 3.78, Ur = 5.84, Ur = 7.52 and Ur = 8.77, respectively. The 2P and 2S vortex modes observed in the current study agree well with those in the DES study [18]. Williamson et al. [32–34] stated that the 2S vortex mode indicates that each half cycle results in single vortex shedding into a wake, whereas the 2P vortex mode indicates that each half cycle results in a pair of vortex shedding. As concluded in a previous study [33], the vortex mode behind the cylinder is affected by the fluctuation in cylinder transverse displacement. At U_r_ = 3.78, the 2S vortex mode is observed, as shown in Fig 10. It is observed that cylinder response is small (Ay/D<0.3) at the initial branch. At peak amplitude or in lock-in region (Figs 11 and 12), pairs of vortices shedding are observed which is resemblance with 2P vortex mode as reported by Williamson et.al [32–34]. The higher displacement response in upper branch is clearly depicted at Ur = 5.84 and Ur = 7.52 in Figs 11 and 12, respectively. At Ur = 8. 77 (Fig 13), which comes under the lower branch, the second vortex is very weak in position and sheds rapidly. The wake pattern and vortex modes justify the 2P shapes in the lock-in region.

## Conclusion

Numerical simulations are conducted to test the performance of the RANS model for a fixed-cylinder case and for a cylinder that is free to oscillate in the transvers direction at Re = 10^4^, mass ratio m* = 11, and ζ = 0.001. This study focuses on the capability of the RANS model to analyze the flow around cylinder at Re = 10^4^, mass ratio m* = 11, and ζ = 0.001. For fixed cylinder case at Re = 10,000, the performances of the mesh and RANS model are tested through 2-dimensional and 3-dimensional numerical simulations. The results are validated with 3-dimensional numerical DES, LES, and experimental results. The fixed-cylinder case reveals that the 2-dimesnsional RANS SST k-w turbulent model can produce acceptable results. However, a high value of C_l_ is obtained due to the low aspect ratio. To investigate the VIV phenomenon, numerical simulations are performed for an elastically mounted rigid cylinder that is free to oscillate in the cross flow direction at U_r_ = 3 to 14 with constant Re (Re = 10^4^) and mass ratio (m* = 11). The 2-dimensional SST-kw results agree well with those of the 3-dimensional DES and experimental studies. The maximum amplitude, A_y_/D = 0.92, is observed at the upper branch, which agrees well with the 3- dimensional DES results. Compared with the DES study and experimental results, the current study observed earlier transition from upper to lower branch. The RANS SST k-w model confirms the formation of different vortex modes at U_r_ = 3 to 14. The agreement with experimental and 3-dimensional computationally expensive DES and LES results builds confidence in using the 2-dimensional RANS SST k-w turbulent model and paves the way for highly complex analyses.
